# Landscape dynamics and diversification of the megadiverse South American freshwater fish fauna

**DOI:** 10.1073/pnas.2211974120

**Published:** 2023-01-03

**Authors:** Fernanda A. S. Cassemiro, James S. Albert, Alexandre Antonelli, André Menegotto, Rafael O. Wüest, Felipe Cerezer, Marco Túlio P. Coelho, Roberto E. Reis, Milton Tan, Victor Tagliacollo, Dayani Bailly, Valéria F. B. da Silva, Augusto Frota, Weferson J. da Graça, Reginaldo Ré, Telton Ramos, Anielly G. Oliveira, Murilo S. Dias, Robert K. Colwell, Thiago F. Rangel, Catherine H. Graham

**Affiliations:** ^a^Department of Ecology, Graduate Program in Ecology and Evolution, Federal University of Goiás, Goiânia 74690-900, Brazil; ^b^Department of Biology, University of Louisiana at Lafayette, Lafayette, LA 70503; ^c^Department of Biological and Environmental Sciences, Gothenburg Global Biodiversity Centre, University of Gothenburg, Gothenburg, Sweden, SE 405 30; ^d^Royal Botanic Gardens, Kew, Richmond, UK TW9 3AE; ^e^Department of Plant Sciences, University of Oxford, Oxford, UK OX1 3RB; ^f^Swiss Federal Institute for Forest, Snow and Landscape Research WSL, Birmensdorf CH-8903, Switzerland; ^g^Federal University of Santa Maria, Rio Grande do Sul, Porto Alegre 97105-900, Brazil; ^h^Pontifical Catholic University of Rio Grande do Sul, Porto Alegre, Santa Maria 90619-900, Brazil; ^i^Illinois Natural History Survey, University of Illinois at Urbana-Champaign, IL 61820; ^j^Federal University of Uberlândia, Minas Gerais, Uberlândia 38400-902, Brazil; ^k^Centre of Biological Sciences, Department of Biology, Graduate Programme in Ecology of In-land Water Ecosystems, Centre of Research in Limnology, Ichthyology and Aquaculture (Nupélia), State University of Maringá, Maringá 87020-900, Brazil; ^l^State University of Mato Grosso do Sul, Mundo Novo 79804-970, Brazil; ^m^Department of Computer Science, Technological University of Paraná, Campo Mourão 87302-060, Brazil; ^n^Department of Systematic and Ecology, Federal University of Paraíba, João Pessoa 58051-900, Brazil; ^o^Department of Ecology, University of Brasília, Brasilia 70910-900, Brazil; ^p^Department of Ecology and Evolution, University of Connecticut, Storrs, CT 06269; ^q^University of Colorado Museum of Natural History, Boulder, CO 80309

**Keywords:** biogeography, lineage diversification, phylogenetic, geological history, tropical biodiversity

## Abstract

Landscape dynamics are widely thought to govern the tempo and mode of continental radiations, yet the effects of river network rearrangements on dispersal and lineage diversification remain poorly understood. We integrated an unprecedented occurrence dataset of 4,967 species with a newly compiled, time-calibrated phylogeny of South American freshwater fishes—the most species-rich continental vertebrate fauna on Earth—to track the evolutionary processes associated with hydrogeographic events over 100 Ma. Net lineage diversification was heterogeneous through time, across space, and among clades. Five abrupt shifts in net diversification rates occurred during the Paleogene and Miocene (between 30 and 7 Ma) in association with major landscape evolution events. Net diversification accelerated from the Miocene to the Recent (c. 20 to 0 Ma), with Western Amazonia having the highest rates of in situ diversification, which led to it being an important source of species dispersing to other regions. All regional biotic interchanges were associated with documented hydrogeographic events and the formation of biogeographic corridors, including the Early Miocene (c. 23 to 16 Ma) uplift of the Serra do Mar and Serra da Mantiqueira and the Late Miocene (c. 10 Ma) uplift of the Northern Andes and associated formation of the modern transcontinental Amazon River. The combination of high diversification rates and extensive biotic interchange associated with Western Amazonia yielded its extraordinary contemporary richness and phylogenetic endemism. Our results support the hypothesis that landscape dynamics, which shaped the history of drainage basin connections, strongly affected the assembly and diversification of basin-wide fish faunas.

## Significance

South America harbors the most diverse freshwater fish fauna worldwide; however, scientists have few clear answers about the origins of this megadiversity. Here, we used the most complete datasets of geographic distributions and evolutionary relationships of South American fishes to date, to track the influence of the geological history on the origins, extinctions, and interchanges of these fishes over the past 100 My. We found that abrupt increases of species origination coincided in time and place with major mountain uplift and river re-arrangement events. Species in Western Amazonia originated faster and persisted longer than those in other regions. Thus, this region acted as a source of dispersal to other regions, enhancing the exceptional diversity of fishes across the entire continent.

Geological and climatic events are widely believed to shape the biodiversity of continental biotas (1–3), yet we are only beginning to understand the nuanced ways in which individual geological and climatic events have contributed to evolutionary diversification (speciation minus extinction) across large spatial scales (4–7). South America harbors the most diverse fauna of continental freshwater fishes in the world (~5,750 species), providing unique opportunities to study the effects of geological history and river dynamics on diversification in obligate aquatic taxa (8, 9). Hydrogeographic processes, operating over tens of millions of years, have caused predictable changes in the geometry of river drainage networks, by isolating and merging portions of adjacent river basins and their connections to the sea, and by altering the physiochemical characteristics of water discharge (10, 11). Here, we evaluate the influence of major geological events on diversity patterns of obligate freshwater fishes of South America over the past 100 Ma, the time period over which hydrogeographic events shaped the origins of modern fluvial systems (4, 5, 12). We conducted the most comprehensive assessment of diversification in this group to date, using an extensive dataset on species geographic occurrences and a newly compiled, species-dense phylogeny of South American freshwater fishes (13). This new synthesis afforded us the opportunity to link unique hydrogeographic events with the spatial and temporal diversification and dispersal of individual fish clades.

**Fig. 1.**
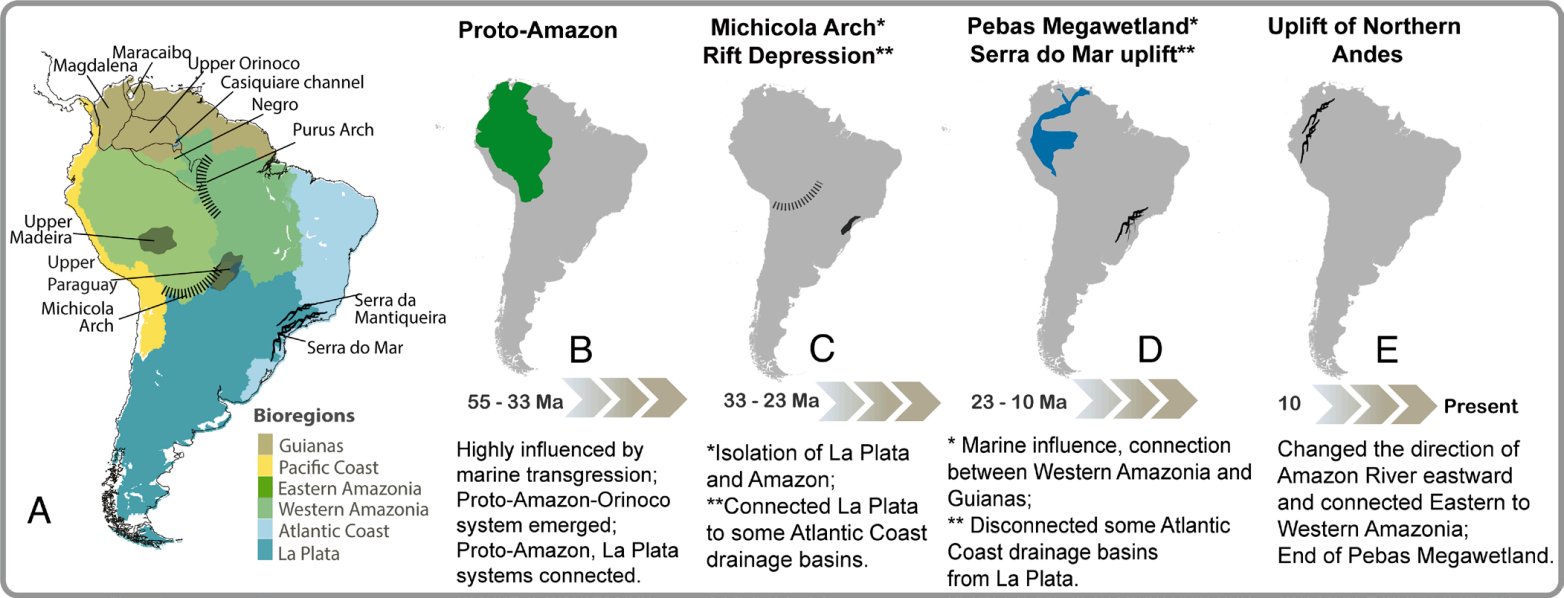
Bioregions, principle current landforms and sub-basins, and approximate chronology and location of the principal landscape evolution events that shaped the current drainage basins of South America and influenced the diversification of freshwater fishes. (*A*) Current river basins and geological formations mentioned in the text and the six bioregions proposed (detailed in *SI Appendix*). (*B*–*E*) Between 100 and 55 Ma, aquatic systems in South America were intermittently connected by multiple marine transgressions and regressions.Thus drainages across the continent during this time were intermittently connected by epicontinental seaways. During this time, the Proto-Amazon basin was the main drainage of northern South America, flowing through the sub-Andean foreland. At the same time, the Paraná and Paraguay basins (La Plata bioregion) represented major aquatic systems in South America (4, 5, 10, 11). Additional information about principal landforms controlling basin connectivity at each time interval and for each bioregion delineation appears in *SI Appendix*, Table S1.

The historical dynamics of South American river basins and aquatic biotas were strongly shaped by four prominent geophysical events (Fig. 1) (11, 14). The first was the final separation of South America from Africa during the Late Cretaceous (c. 100 Ma). Between the Late Cretaceous and Early Paleogene (c. 100 to 55 Ma), river drainage patterns of South America were controlled by the location of the preexisting continental uplands (cratons and shields), ongoing uplift of the Andean cordilleras, super greenhouse climatic conditions characterized by high temperatures and precipitation, and dramatically fluctuating eustatic sea levels. As a result, most low-elevation coastal plains and interior structural basins were covered by nearshore marine habitats, and upland freshwater riverine and riparian habitats were intermittently isolated and connected (4, 15). During the Paleogene (c. 55 to 33 Ma), the Proto-Amazon-Orinoco river basin (Proto-Amazon basin, hereafter) drained the Sub-Andean Foreland basin, including much of northern South America and the northern La Plata region (Fig. 1 *A* and *B*; 5, 16).

Second, intraplate compression and tectonic subduction along the Pacific margin during the Oligocene (c. 33 to 23 Ma) drove tectonic uplift of the Altiplano and Michicola Arch (c. 30 Ma) associated with formation of the Bolivian Orocline (17). These orogenic deformations intermittently isolated and connected rivers among the Western Amazonian, Upper Madeira, and Upper Paraguay sedimentary basins of the Sub-Andean Foreland, facilitating vicariance and biotic exchanges across their watershed divides (Fig. 1*C*; 4, 18–20). Third, tectonic reactivation and uplift of Serra do Mar and Serra da Mantiqueira ranges in southeastern Brazil during the Early Miocene (c. 23 to 16 Ma) re-routed some rivers from the La Plata basin directly to the Atlantic (Fig. 1*D*; 21, 22–24), isolating many terrestrial and aquatic species in the coastal basins of the Atlantic Forest. Also, at about this time (c. 23 to 10 Ma), the Pebas Megawetland extended over large areas of the modern Western Amazonia and Orinoco basins (Fig. 1*D*; 5, 7, 22–26). Fourth, the uplift of the Northern Andes during the Late Miocene and Pliocene (c. 10 to 4.5 Ma), which profoundly reorganized regional river drainage networks, isolated the modern Amazon, Orinoco, Magdalena, and Maracaibo river basins and connected the modern Western and Eastern Amazon basins, thereby forming the modern transcontinental Amazon River (Fig. 1*E*; 16, 27).

Variation in connectivity and configuration of regional river networks resulting from these four major geological events strongly shaped diversity patterns of the Neotropical freshwater fish fauna (4, 12). In particular, river capture, in which a river drainage system is diverted from its historic bed to a neighboring bed, is a landscape evolution process that exerts a potent influence on diversification in obligate freshwater organisms, because it both severs existing and constructs new corridors of aquatic habitat among portions of adjacent drainage basins (18, 28). Because continental fishes are eco-physiologically restricted to freshwater habitats within drainage basins, watersheds represent natural dispersal barriers, as evidenced by the strong spatial concordance of geographic ranges in freshwater fish species with basin boundaries (28, 29). By isolating and connecting populations of aquatic taxa across watershed divides, river capture exerts complex effects on the diversity of freshwater organisms, for example by elevating extinction risk through geographic-range contraction, promoting speciation by genetic isolation and vicariance, and increasing biotic homogenization by dispersal and gene flow (16, 30–32).

**Fig. 2.**
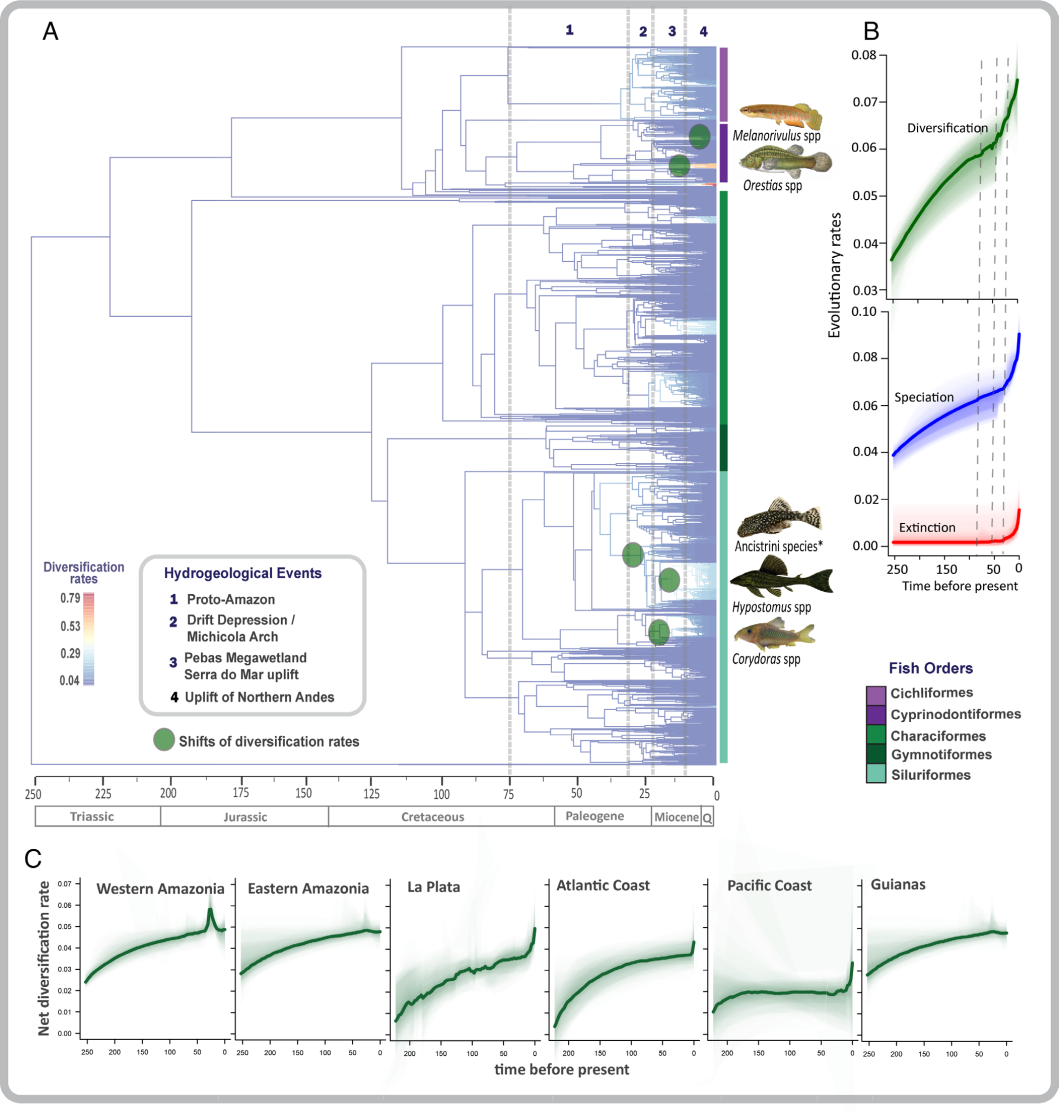
Changes in the rates of net lineage diversification among South American freshwater fishes. Tips represent 2,523 fish species. (*A*) Branch colors indicate net lineage diversification rate estimated by BAMM, where red indicates highest and blue lowest diversification rates. Significant shifts in diversification rates are shown as pale green circles on the branches. Selected representative clades of *Melanorivulus*, *Orestias,* Ancistrini, *Hypostomus*, and *Corydoras* species are illustrated. The principal orders are represented by colored columns to the right of the tree tips. The timescale at the bottom is expressed in millions of years ago (Ma). Vertical dashed lines indicate timing of the main principal hydrogeographic events detailed in the inset legend on the left. (*B*) Rates-through-time plots based on BAMM estimations, considering all bioregions together (see *Material and Methods* for parametrization details). The shaded areas around the curves correspond to 95% CIs of the estimated rates. Dashed lines indicate the time period when most of shifts in diversification rates were estimated. (*C*) Rates-through-time plots considering the species present in each bioregion separately. Rates of diversification, speciation, and extinction were estimated mainly within crown taxa. The five photographs: Wikipedia Commons. *Ancistrini species of genera *Hopliancistrus, Guyanancistrus, Pseudolithoxus, Lasiancistrus, Pseudancistrus, Panaque*, and *Pterygoplichthys.*

Although the role of geological events in shaping the evolution of rivers and freshwater diversity has long been recognized, the relative contributions of particular geological events remain poorly understood. Insights into their contributions can be gained only by studying diversity patterns at appropriate spatial, temporal, and taxonomic scales (28, 33–36). For instance, recent studies identified Western Amazonia as the center of Amazon fish diversity (high species richness, low phylogenetic diversity (PD), and high phylogenetic clustering, compared to Eastern Amazonia), with younger fish lineages dispersing progressively eastward across Amazonia after the formation of the modern transcontinental river c. 10 Ma (37–39). However, this interpretation did not consider the more ancient history of Neotropical fishes in the upland Brazilian and Guianas Shields, the formation of the modern lowland (< 250 m.a.s.l.) fauna in the Proto-Amazon basin, and the phenotypically and taxonomically modern composition of all the known Miocene paleo-ichthyofaunas (4, 40). Taking this deeper history into account, Pliocene and Pleistocene events may have served more as buffers against extinction than as drivers of speciation in the formation of Amazonian fish species diversity (Fig. 2; 15, 41). In fact, the most species-rich clades of Neotropical freshwater fishes are thought to have radiated during the Paleogene (c. 63 to 23 Ma) (4, 42, 43).

In the case of South American freshwater fishes, previous macroevolutionary studies have been hindered by the large number of species (~5,750), remote sampling localities, and logistical difficulties of gathering reliable data (8). Our new data on fish distributions, which we combine with a new, time-calibrated molecular phylogeny, offer powerful resources to study the role of geomorphological events and associated river captures in shaping fish diversity over longer time periods and larger spatial scales than has previously been attempted. In particular, we evaluate the prediction that the high diversity in Western Amazonia was influenced by biogeographical bridges formed across different aquatic systems and time periods, which led to both accelerated diversification rates and a role for Western Amazonia as a principal source of freshwater fish species for all of South America.

## Results and Discussion

### Diversity Patterns Are Associated with Landscape Evolution.

Here, we used a new and unprecedentedly complete occurrence dataset of South American fishes, comprising approximately 95% of all fish species described for the continent, and a newly compiled time-calibrated molecular phylogeny of all Neotropical freshwater fishes (13). We calculated species density in each of level 5 sub-basins (44; see *Material and Methods*) in South America from collection locality data (13) and estimated lineage diversification rates and rate shifts through time (36) based on phylogenetic data. Finally, we integrated occurrence and phylogenetic data to estimate clade ancestral areas, species dispersal events, and phylogenetic endemism (PE). Our approach explores associations between landscape evolution events (e.g., tectonics, erosion, sea-level changes) and patterns of net lineage diversification (speciation minus extinction) and biogeographic dispersal (i.e., changes in species’ geographical ranges).

Basins were defined using a high-resolution SRTM digital elevation model (44). We conducted ancestral area estimation of freshwater fishes using the Bayesian biogeographical inference model [BAYAREALIKE (45); see *Material and Methods*; SI *Appendix,* Table S2] implemented by software platform BioGeoBEARS (46, 47) to estimate geographic-range change events among six bioregions delineated using taxonomic and geographic criteria (*SI Appendix*, *Biogeographical Regionalization* and Fig. S1). Other commonly used models (DEC and DIVALIKE) varied in the number of dispersal events estimated, but all showed the same patterns of dispersal; thus, our results are robust to method (SI *Appendix,* Tables S3–S7). We interpreted the underlying processes revealed by these biogeographical reconstructions, taking into account known events of expansion, contraction, and connectivity of continental drainage basins. As fish dispersal is limited by drainage basin boundaries, it is important to reconstruct dispersal events to understand how and when diversification processes occurred.

We found patterns of species density and PE were strongly associated with the time and place of specific landscape evolution events, which are interpreted to have both constrained and promoted species interchanges across basins over millions of years. Most dispersal events among the bioregions occurred from Western Amazonia to the La Plata bioregions, facilitated by intermittent connections across the low-elevation watershed (Izozog wetlands) between the Upper Madeira and Upper Paraguay basins. During the Late Miocene, uplift of the Northern Andes and Vaupés Arch resulted in the Amazon River breaching the Purus Arch, connecting the Western and Eastern Amazonian basins and increasing species interchange between these two large, low-elevation and species-rich bioregions. In addition, some basins along the Atlantic Coast have been isolated for the last 25 Ma, contributing to high fish endemism of this bioregion. Biogeographical corridors between Western Amazonia and other bioregions were fundamental in the accumulation of fish diversity across tropical South America. Details of these diversification events are discussed below.

### Diversification Patterns.

We found that the diversification of freshwater fishes in South America was heterogeneous through time, across space, and among taxonomic groups (Fig. 2). Diversification results based on Bayesian analysis of macroevolutionary mixtures [BAMM; (48) – see *Material and Methods* for details] for 2,523 species revealed five rapid shifts in diversification rates—two during the Early Paleogene (~30 to 23 Ma) and three during the Miocene (~20 to 7 Ma) (Fig. 2 *A* and *B*). These changes in diversification were primarily driven by shifts in three clades of catfishes (the tribe Ancistrini and the genera *Hypostomus* and *Corydoras*), all armored catfishes (Loricariidae), and the most diverse family of South American fishes (see ref. 49 and *SI Appendix*, Fig. S2). The most recent rapid diversification shifts were in two clades of small-bodied toothcarps (Cyprinodontiformes): *Orestias* with about 45 species restricted to high-elevation (>1,000 m.a.s.l.) lakes and rivers in the Andes (50, 51), and *Melanorivulus* with about 60 species, principally distributed in rivers draining the Brazilian Shield, in the La Plata and Southeastern coastal Atlantic basins (52) (*SI Appendix*, Fig. S2).

Rates of diversification, especially for the species-rich clades Ancistrini, *Hypostomus,* and *Corydoras*, began to accelerate about 30 Ma (Fig. 2*A*). At first, the rise of Michicola Arch (c. 30 Ma) blocked the connection between the Western Amazonia and La Plata bioregions (4). Subsequently, the formation of the Pebas Megawetland (c. 23 to 10 Ma) established a large sediment-rich (whitewater) floodplain-like habitat connection between the Western Amazonia and Orinoco basins (5, 14, 15). At this time, the Purus Arch blocked dispersal between Western and Eastern Amazonia, isolating these bioregions from c. 40 to 10 Ma (4, 5), with the highest peak in situ diversification rates around 30 Ma (Fig. 2*C*). Throughout most of the Paleogene and Early Neogene (50 to 10 Ma), the region of the Proto-Amazon basin was largely interconnected, but subjected to retraction and expansion of lowland freshwater habitats caused by extensive marine transgressions and regressions (4, 5, 42). These dynamics likely promoted net diversification in obligate freshwater taxa by episodically isolating and merging riverine and riparian habitats (34, 42, 53, 54), leading to high contemporary evolutionary distinctiveness (the amount of unique evolutionary history a lineage represents), especially in basins located along the Amazon River and in the southernmost basins in the La Plata bioregion (*SI Appendix*, Fig. S3). Paleontological data corroborate the hypothesis that Paleogene radiations of freshwater fishes occurred in the Proto-Amazon basin (40, 42, 54). Subsequently, the relatively rapid uplift of Northern Andean Cordilleras (c.11-9 Ma) caused drastic changes in drainage networks (55, 56). These tectonic events changed the direction of the Amazon River flow from northward to eastward and connected the Western and Eastern Amazonia bioregions without notably increasing rates of net diversification (Fig. 2*C*). Combined, these hydrogeological events likely resulted in Western Amazonia having the highest diversification rates in South America (Fig. 2*C*). Finally, high speciation rates over the last 10 My could have been influenced by uplift of the Serra do Mar and Serra da Mantiqueira ranges, which served to isolate the La Plata basin from some Atlantic Coast drainage basins, associated with a net diversification increase by vicariance and geodispersal (Fig. 2 *A*–*C*).

### Routes and Timing of Dispersal.

**Fig. 3.**
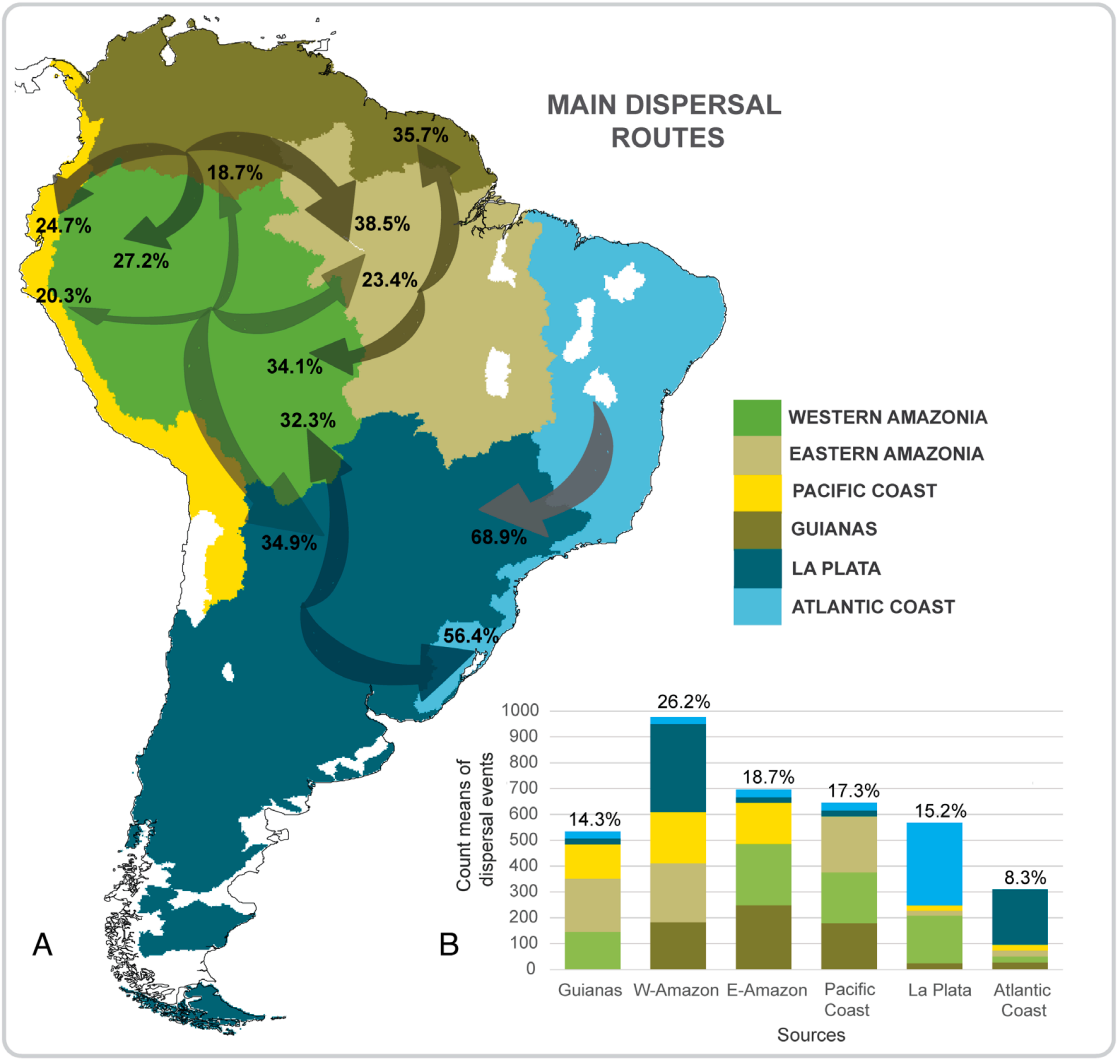
(*A*) Proportions of biogeographical dispersal events among bioregions. Arrows indicate dispersal directions, and numbers represent percentage of the mean (of total 20 simulations) for all dispersal events from source to sink bioregions. Width of arrows is proportional to the number of dispersal events. (*B*) Total count means of dispersal events from source (axis x) to destination bioregions (colored legend). Percentages over each column represent the relative contribution of each source bioregion to total number of dispersal events. The drainage basins in white were not considered in the study for lack of adequate sampling effort. For more details regarding dispersal analysis results, see *SI Appendix*, Tables S5–S7.

We recorded a total of 3,730 estimated fish dispersal events across South America, mostly by anagenesis—that is, by geographic-range expansion decoupled from speciation (43). Our biogeographical analyses show Western and Eastern Amazonia together were the source of nearly half (44.8%) of all estimated dispersal events (Fig. 3*B*). Western Amazonia, especially during the Miocene, was the most important source of freshwater fish lineages among the six bioregions considered here (Fig. 3 *A* and *B* and *SI Appendix*, Tables S5–S7) (26.2% of all dispersal events; Fig. 3*B* and *SI Appendix*, Table S5), with the greatest number of dispersal events (34.9%) to the La Plata bioregion (Fig. 3*A* and *SI Appendix*, Table S7). The Eastern Amazonia bioregion was the next-most -common source (18.7%; Fig. 3*B*), with most of these dispersal events to the Western Amazonia and Guianas bioregions (34.1% and 35.7%, respectively; Fig. 3*A* and *SI Appendix*, Table S7).

**Fig. 4.**
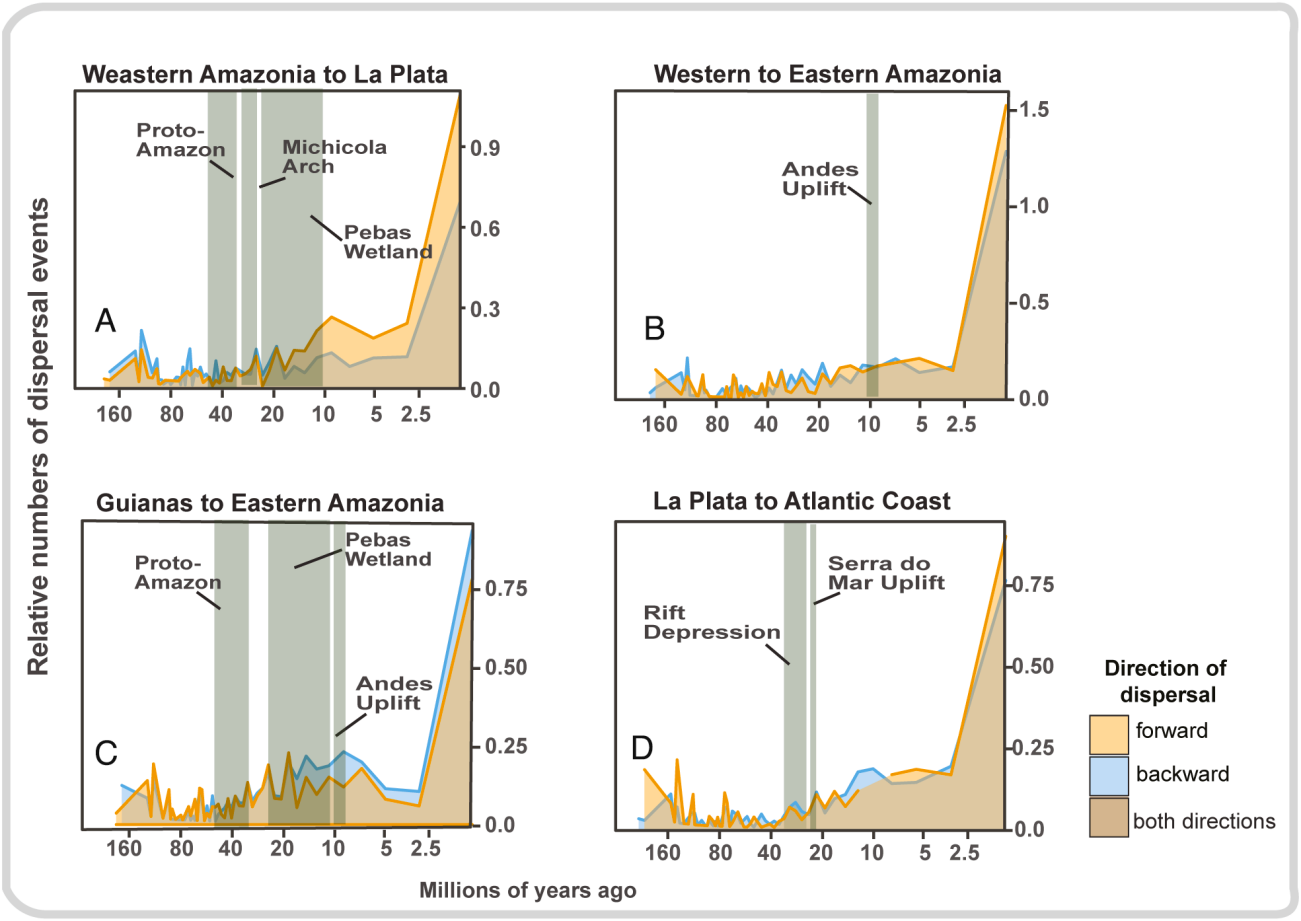
Principal dispersal events through time among the bioregions that acted most prominently as species sources and geological events that influenced the connections among drainage systems, triggering principal dispersal events across South America. We depicted, here, only dispersal events since 160 Ma, but older events were included in the total calculation of events. The hydrogeographic events considered follow those represented in Fig. 1. The relative number of dispersal events is the total number of dispersals divided by the log-number of lineages within each time bin (1 My). (*A*) Dispersal events between Western Amazonia and La Plata bioregions, (*B*) between Western and Eastern Amazonia, (*C*) Guianas and Eastern Amazonia, and (*D*) La Plata and Atlantic Coast. The timescale on the X-axis is logarithmic.

In addition, we estimated the relative probability of dispersal among bioregions through time, from 260 Ma to present, based on the principal hydrogeographic events that we hypothesized were important (Fig. 1 and *SI Appendix*, Table S1 for more details). The most important events occurred from 55 to 0 Ma (see Fig. 1 and *SI Appendix*, Table S1) resulting in three important peaks in dispersal from the Western Amazonia to the La Plata bioregion (Fig. 4*A*): about 30 to 25 Ma, around 20 Ma, and approximately 10 Ma. The timing and location of these dispersal peaks suggest a causal connection with megariver capture events among structural basins of the sub-Andean foreland (18, 26). In addition, dispersal from the Eastern to the Western Amazonia bioregion peaked between 10 and 7 Ma and was slightly greater than dispersal from Western to Eastern bioregions during the same period (Fig. 4*B*). The timing and location of this dispersal peak indicate a causal connection with the megariver capture event that formed the modern transcontinental Amazon River (38). Finally, dispersal between Western and Eastern Amazonia increased substantially after 5 Ma in both directions (Fig. 4*B*), continuing into the present, beginning with the dramatic increase in Amazon River sediment flux and várzea habitat during the Pliocene (15).

Although the history of landscape connections between the Western Amazonia and La Plata basins is not entirely clear, they likely involved multiple river capture events over millions of years (4, 15). The fish faunal interchange around 30 Ma between these bioregions (Fig. 4*A*) may have been enhanced by megariver capture events (>10,000 km^2^) in the sub-Andean foreland in the vicinity of the Bolivian Orocline (16, 28, 29). The abrupt decrease in dispersal between these bioregions coincides temporally with the rise of the Michicola Arch (c. 30 to 23 Ma; Fig. 4*A*), an important dispersal barrier for riverine fishes (57). The beginning of the second peak of dispersal coincides with the origin of the Pebas Megawetland (c. 23 to 10 Ma; Fig. 4*A*), although there is no geological evidence that the Western Amazonia and La Plata bioregions were connected during this time (58). Further, even though the La Plata bioregion has not been permanently connected to Western Amazonia for at least 10 Ma, a semipermeable dispersal corridor between the two systems emerges, to this day, when intense, seasonal floods permit movement of species adapted to ephemeral stream and marsh environments (59). This ephemeral connection explains at least some of the most recent dispersal events (60; Fig. 4*A*), supporting the view that La Plata harbors more clades from the Amazon basin than vice versa (61–63). It also provides quantitative evidence that similarities in species composition between Amazon and the headwaters of La Plata are due to connections that predate the formation of the modern Amazon watershed (61). Other important past drainage connections are highlighted by our results, including an increase in the frequency of dispersal events from Eastern to Western Amazonia (Fig. 4*B*). This increase coincided in time with the most recent uplift of the Northern Andes, starting c. 10 Ma, which changed the direction of Amazon River flow and connected these two bioregions.

With the formation of the transcontinental Amazon River c. 10 Ma (4, 5, 16), fishes long adapted to the highly productive whitewater floodplains and river channels of the sub-Andean foreland were able to disperse into Eastern Amazonia and colonize clearwater rivers of the Brazilian and Guiana Shields (Fig. 4*C*). The formation of the largest waterway in the world likewise allowed reciprocal dispersal of fishes from the clearwater shield rivers to the Western Amazonia lowlands (38). The most recent uplift of the Northern Andes (c. 10 Ma) created a divide between the upper Amazonas and Orinoco drainage systems, which explains the subsequent decrease in dispersal. However, this was a semipermeable divide, with a dispersal corridor through the Casiquiare channel allowing some fish species to move between the Upper Negro and Upper Orinoco basins (59). Previous studies have suggested that the lineages within the Orinoco and Amazon basins appear to share common ancestors more recently than 10 Ma (64, 65). Loricariid (66) and cichlid (67) species, currently distributed in both basins, are likely examples of dispersal between these regions.

**Fig. 5.**
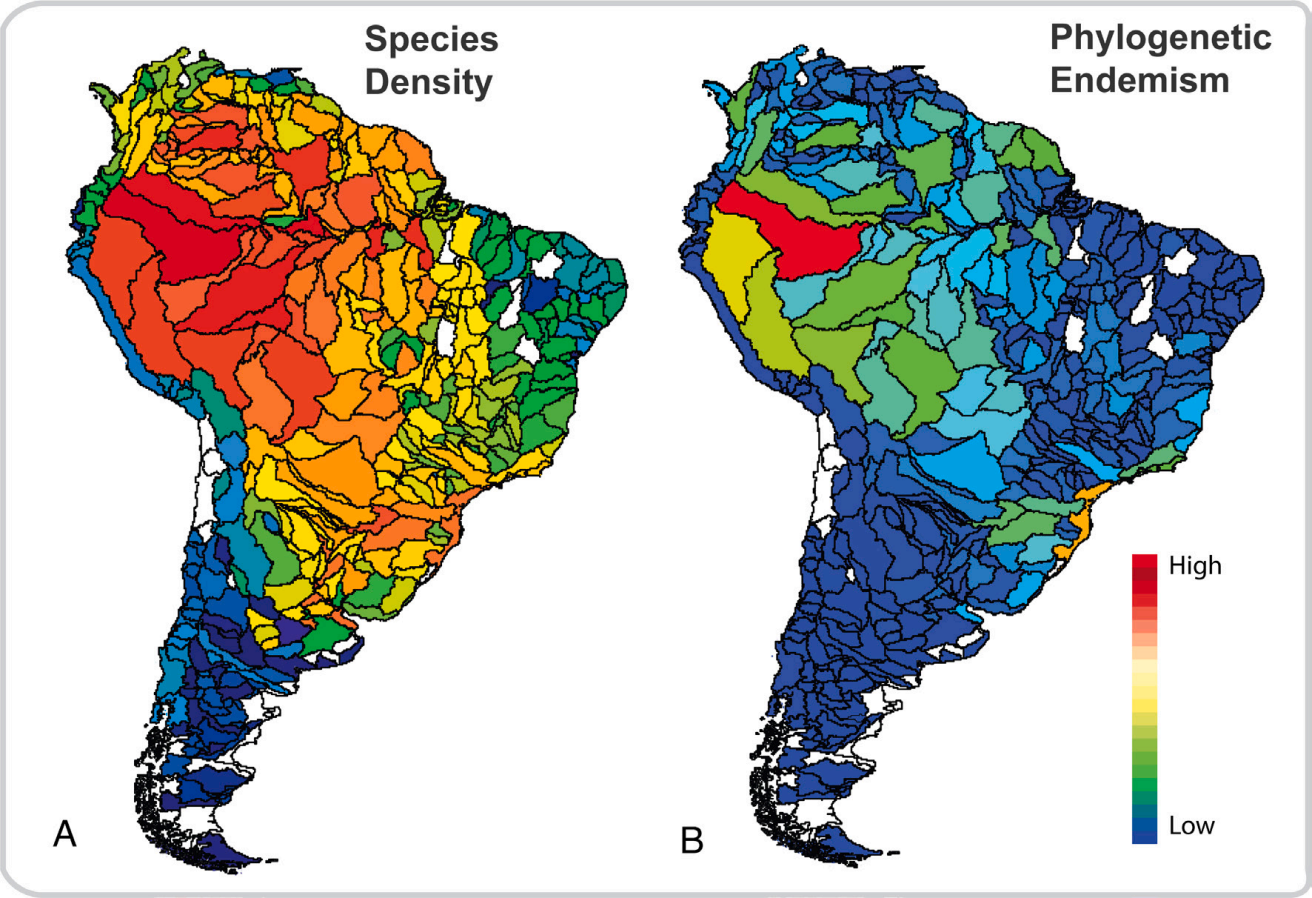
(*A*) Species-density map for 4,967 freshwater fish species. Density was calculated as Species/(Area)^b^, where the value of *b* was calculated using the Species–Area relationship (Species is the total number of species in a basin, and Area is the surface area of the basin). (*B*) PE (mean) for each drainage basin. PE for 2,523 species, a combination of the PD and weighted endemism measures, identifies bioregions to which unique phylogenetic lineages are restricted. The drainage basins in white were not considered in the study for lack of adequate sampling effort (see *Methods* for details).

Dispersal between the large interior La Plata and Atlantic Coastal basins occurred because of a new connection between the two regions, as a result of rift depression during most time periods, but intensified after 30 Ma (Fig. 4*D*). These connections had a strong impact on dispersal events, promoting diversification and high levels of endemism (68, 69; see also Fig. 5*B*). A concentration of dispersal events from the interior to the Atlantic Coast basin during this period is also observed in the northeastern Brazil region (21, 70). Some fish fossils from large rivers of the La Plata bioregion are found in the Tremembé Formation (21), a sedimentary formation in the Ribeiro de Iguape Basin of the Atlantic Coast. Subsequent to the uplift of the Serra do Mar, most Atlantic Coast basins flowed directly to the sea, with dispersal corridors between these two bioregions persisting only among the basins located in the northeast of Brazil (21). This pattern explains the high number of dispersal events from the La Plata basin to the Atlantic Coast bioregion (from c. 20 Ma to the present). The scenario of Miocene connections between the La Plata and Atlantic Coast bioregions is supported by the presence of several extant species distributed on both sides of the contemporary watershed divide (71, 72).

### Contemporary Species Density and PE Patterns interpreted in Light of Historical Events.

Freshwater fish species richness per area (mapped as species density) showed clear latitudinal and longitudinal patterns across South America. As in many other taxa, fish species richness (corrected for the area of the basin, see *Methods*) is lower at higher latitudes and higher near the Equator (Fig. 5*A*). The species richness and endemism values of freshwater fishes in Western Amazonia are extremely high (Fig. 5 *A* and *B*), increasing in the opposite direction of the river flow (37). Marine transgressions since the Late Cretaceous likely reduced species richness near the mouths of most lowland Neotropical rivers (16). The decreasing pattern of diversity from west to east reflects a pattern reported for many terrestrial groups, for both macro- and microorganisms (5, 73), but differs markedly from the general pattern observed in rivers worldwide, described as the river continuum concept, in which diversity increases from headwaters to mouth (74).

A lengthy history of geographical isolation contributed to explaining the high level of PE observed in the basins of the southeastern Atlantic Coast (Fig. 5*B*). The isolation of some Atlantic Coast basins from La Plata basin, driven by the uplift of the Serra do Mar, provided stable relictual habitats and opportunities for vicariance across higher upland elevations. These areas have recently been described as historically stable refugia for diverse taxa in the Atlantic Forest (75, 76), which holds high levels of genetic diversity and endemism for several groups (21, 77, 78).

## Conclusions

Advances in the biogeography of South American fishes have been hampered by poor sampling and a focus of study within a limited geographic focus, especially in the Western Amazon, Guianas Shield, and southeastern Brazil (34, 43, 79, 80). Here, we present a continental-scale study using a newly complied, synthetic phylogeny of the entire fauna of South American fishes and a newly assembled dataset of geographic distributions. We minimized previous sampling biases by employing a new simulation procedure to estimate species presence/absence data in each sub-basin, following a statistical methodology that accounts for uncertainty and uneven sampling. These geographic distributions enable a more reliable evaluation of the relationships between landscape evolution and freshwater fish diversity. A further analytical challenge is to quantify the impact of phylogenetic uncertainty on estimates of rates of diversification. Here we employed the most comprehensive dataset on South American fish distribution and phylogeny available today. Still, future extensions of taxonomic coverage of molecular phylogenies and species geographic distribution should further reduce residual uncertainty of our results.

Our study shows that substantial shifts in lineage diversification rates predate the Late Neogene uplifts of the northern Andes and were not confined to the area of the modern Amazon basin. Rather, a combination of biogeographic and phylogenetic evidence indicates that many fish lineages that are now distributed over much of tropical South America originated in Western Amazonia during the Paleogene (34, 43). Our results reinforce the view that landscape dynamics occurring over at least 100 My established the modern drainage basins that acted as semipermeable dispersal corridors and barriers, contributing to the assembly of modern basin-wide freshwater faunas (11, 15, 80). Hydrogeographic events caused the isolation and mixing of preexisting aquatic faunas through a series of vicariance and dispersal events, resulting in a complex history of net diversification and dispersal among river basins. Western Amazonia differs from other regions in presenting exceptionally high species density and PE, acting as both a museum and a cradle of diversity (39, 81) and representing an important source of species for other regions of the Neotropics.

In recent decades, alarming rates of deforestation, land-use degradation, river damming, and water pollution in South America (82, 83) threaten many aquatic species, especially in southern portions of the Amazon basin and the Atlantic Forest (84, 85). As with all aspects of biodiversity, documenting the evolutionary and ecological processes underlying the origins and maintenance of South American fishes offers fundamental guidance in efforts to conserve this unique diversity, the most species-rich continental vertebrate fauna on Earth.

## Materials and Methods

### Occurrence and Phylogenetic Data.

We compiled 306,425 occurrence records of 4,967 freshwater bony fish species in South America through an extensive search of the literature, museums, FishBase (http://www.fishbase.org/), FishNet (http://www.fishnet2.net), Global Biodiversity Information Facility (GBIF) (http://www.gbif.org/), SpeciesLink (http://splink.cria.org.br), and Datos de Peces de Aguas Continentales de Argentina (http://www.pecesargentina.com.ar/base_peces/inicio_busq.html). We automated the process of data filtering with code that considered a suite of common possible errors in those databases (e.g., georeferencing errors, synonymies, collection year, typographic errors, exotic species). We removed all records of species that are exotic to South America, based on Gubiani et al. (86). Species names were validated following the Catalog of Fishes of the California Academy of Sciences (http://www.calacademy.org/scientists/projects/catalog-of-fishes) and carefully revised by taxonomic specialists in Neotropical fishes. Occurrence records were then mapped into 490 drainage basins (the lowest level unit used in our analysis), as delineated by the HydroBASINS database, a subset of the HydroSHEDS project (level 5, hydrological data and maps based on Shuttle Elevation Derivatives (44), available at https://hydrosheds.org/page/hydrobasins), to estimate spatial patterns of species richness and diversification rates across South America. The HydroBASINS database provides a range of drainage basin resolutions, from level 1 (low) to 12 (high resolution). We chose level 5 as an intermediate resolution that appropriately matches the spatial scale of sampling effort within sub-basins. A species-density (richness per area) map was created (Fig. 5), controlling for the surface area effect of each basin on species richness.

We used a newly compiled, comprehensive, time-calibrated phylogeny of Neotropical freshwater fish taxa based on DNA sequence studies, generated by dozens of published papers over three decades by the ichthyological research community and all available at GenBank (Fig. 2*A*; treefile available online at ref. 13). We assembled a supermatrix comprising 43,850 bp from 51 independently aligned and trimmed markers, including 5,984 terminal taxa representing 3,169 species (51%), and 861 genera (99%) for the entire Neotropical freshwater fish fauna. We performed tree searches in RAxML assuming independent GTR + G models for partitions and a start tree with a few justifiable node constraints. After randomly sampling one specimen per species, we estimated divergence times using “Congruification,” a tool for time-scaling large trees using secondary calibrations from a reference time-calibrated tree, which included 31 fossil calibrations from time-calibrated phylogenies of Actinopterygii (87) and Potamotrygonidae (53). The species phylogenetic tree (3,169 species) was compared to the species occurrence list (4,967 species), and the 2,523 species that were present in both datasets were used in all evolutionary analyses. Fossil records were not included in the analyses because the paucity of detailed studies of morphological characters of fossils, especially for Amazonian fishes, allows only the identification of coarse taxonomic levels. This deficiency limits potential use of Neotropical freshwater fossil fishes in biogeographic surveys.

### Filling the Gap in Species Distributions.

The mapping of species records in drainage basins revealed a pattern of heterogenous sampling effort across South America, with as much as a tenfold difference in number of occurrence records and fivefold difference in species richness in basins of comparable area. To mitigate sampling heterogeneity, we employed a novel simulation procedure to estimate species presence/absence given sampling effort biases, following principles of existing methodology that accounts for uncertainty. The approach consists of two iterative steps, which we repeated independently for each focal basin. We first calculated a completeness index for each drainage basin (level 5) (88, 89), by estimating the probability that the next record would add a new species to those recorded in the basin (the “coverage deficit” of ref. 71). If the lower 95% confidence limit of the completeness index was greater than or equal to 0.75, and the number of unique records (i.e., unique combination of species name, geographic coordinates, and sampling date) was larger than 50, the sampling effort at the focal drainage basin was considered acceptable. However, if the completeness index was less than 0.75, we randomly sampled an occurrence record among all adjacent drainage basins, i.e., level 5 basins in the same larger catchment delineation (at level 4). We adopted this conservative criterion because random effects may result in an unstable completeness estimate in basins with small numbers of records, returning artificially high completeness values (89). Also, resampling from adjacent basins that belong to the same large basin (level 4) conservatively assumes low dispersal capacity and does not increase the potential species pool. The procedure then iterates back to step one, sampling again, with replacement, after each new occurrence record is inserted into the focal basin, until the completeness index reaches at least 0.75. We repeated this procedure 100 times for all South American drainage basins to account for uncertainty, thereby generating 100 matrices of species presence/absence. Further, by working with level 5 basins, we reduced errors of omission and commission in the species occurrence dataset. At a finer resolution (i.e., level 6 basins), it is not possible to infer patterns of species density due to insufficient occurrence records and at a coarser resolution (i.e., level 4 basins), the capacity for interpretation is reduced. We excluded from analysis 20 basins that were poorly sampled (white basins in Fig. 5) but did not have an adjacent basin (i.e., an additional level 5 basin within level 4 catchment bioregion; Species records and richness summary per basin are available at ref. 13). By carefully evaluating sampling effort issues, we believe that our knowledge of this fish fauna is now relatively robust. Additional studies addressing this topic have also concluded that modern taxonomic and geographic datasets of South American fishes are now sufficiently robust against the vagaries of taxonomic practice to conduct faunal-scale assessments of biodiversity and conservation (e.g., refs. 90–93).

### Analysis of Phylogenetic Endemism.

We used the pruned phylogeny (2,523 species) to estimate the PE (94) of each drainage basin. PE uses the branch length and the geographic range of the extant descendants of a clade to apportion PD across the areas where it occurs, thereby identifying concentrations of spatially restricted PD (95, 96). See *SI Appendix* for more details.

### Evolutionary Rates.

Diversification, speciation, and extinction rates were estimated using BAMM (48), which is considered an accurate approach among different evolutionary tests [see ref. 97]. However, given the known limitations of BAMM (*SI Appendix*), we also calculated diversification patterns among lineages using the inverse equal splits method (DR; 98), which estimates the splitting rate from each extant species to the tree root without a formal parametric model. As BAMM and DR outcomes were highly correlated (rho > 0.5; *SI Appendix*, Fig. S4), we used rates estimated by BAMM as the main results and treated DR as supplementary. We performed BAMM with three runs each with 1 million Markov chain Monte Carlo (MCMC) generations and a sampling frequency of 1,000. We discarded a burn-in of 10% and confirmed that effective sample size values were appropriate. Results were analyzed and plotted with various functions in the R library BAMMtools, as follows. We derived prior settings using the function setBAMMpriors from BAMMtools before the analysis and modified the default setting to achieve convergence. The priors used were expectedNumberOfShifts = 1.0; lambdaInitPrior = 0.03786; lambdaShiftPrior = 0.85176; muInitPrior = 0.03786; with prior of the time mode being time-variable (vs. time-constant). The prior parameterization ensures that the prior density on relative rate changes across the tree is invariant to the scale of the tree (97). We used computeBayesFactors to identify the best-supported model of rate shifts in our data. We plotted the maximum a posteriori probability shift configuration (getBestShiftConfiguration), the 95% credible set of distinct rate shift configurations (credibleShiftSet), and a "phylorate" graph showing mean marginal posterior density of diversification rates (plot.bammdata) for each lineage (Fig. 2*A*). Rates-through-time plots were also generated for speciation (λ), extinction (µ), and net diversification (*r*) using “PlotRateThroughTime.”

### Ancestral Range Reconstruction and Dispersal Events.

We reconstructed the biogeographic history of freshwater fishes, considering the delimitation of six regions of South America that broadly correspond to regional drainage basins (level 2 of HydroBASINS; see details of bioregions delimitation analysis in *SI Appendix*). To infer geographical range evolution of lineages (i.e., ancestral range analyses; see *SI Appendix* regarding the evolution of ancestral ranges), we applied a dispersal-extinction-cladogenesis model (DEC), Bayesian biogeographical inference (BAYAREALIKE) (45), and maximum likelihood versions of dispersal-vicariance analysis (DIVALIKE) (99) implemented in BioGeoBEARS (46, 100), which also allows the addition of a founder-event parameter *j* in DEC models. The parameter *j* allows that, during cladogenesis, one of the descendants can jump to a new range outside that of the ancestral range without prior range expansion (i.e., dispersal across a barrier; 48,101). Considering that fish species need connections among rivers/basins for dispersal, the parameter *j* was not used in our analysis. Moreover, critiques have been raised on the use of the founder-event parameter *j* in DEC models (101).

Model comparisons, based on the Akaike Information Criterion (AIC) and the small-sample-corrected Akaike Information Criterion (AICc), consistently favored the BAYAREALIKE model *SI Appendix*, Table S2). All models contained parameters *d* (rate of range expansion) and *e* (rate of range contraction). We analyzed dispersal events by taking into account biotic connectivity among all bioregions. Connectivity was modeled based on paleogeographic and geological events (Fig. 1; 4, 5, 16), incorporating time-stratified dispersal multiplier matrices in the model (*SI Appendix*, Table S1). We divided the hydrogeographic history of South American freshwater fishes, from 255 Ma to present, into five strata: 255 to 33, 33 to 23, 23 to 10, 10 to 5, and 5 Ma to present, according to the principal hydrogeological events mentioned in Fig. 1 (see *SI Appendix*, Table S1 for more details). The dispersal multiplier matrices for each of these strata allow estimation of the relative probability of dispersal between bioregions for each time stratum and are roughly scaled to represent the connectivity between the areas during each time stratum. We computed the absolute number of dispersal events through time by extracting the areas and ages of all nodes from the phylogenetic tree, using the Biogeographical Stochastic Mapping approach (100). As the number of lineages (branches) in any phylogeny tends to increase through time, increasing the number of dispersal events toward the present even under a constant dispersal rate, we calculated the relative numbers of dispersal events by dividing absolute numbers by the total number of all lineages within each time bin. However, there are extremely few branches toward the tree root, which causes an over-representation of (relative) dispersal toward the root. To reduce the impact of that bias, the number of dispersals per time bin was divided by the log-number of lineages (per the same time bin).

Additional description of the methodology used appears in *SI Appendix*.

## Supplementary Material

Appendix 01 (PDF)

## Data Availability

Occurrence and Phylogeny data have been deposited in Zenodo (https://zenodo.org/badge/latestdoi/486714511).
