# Supplementary material for: Landscape dynamics and diversification of the megadiverse South American freshwater fish fauna
Source: Proc Natl Acad Sci U S A. 2023 Jan 3;120(2):e2211974120. doi: 10.1073/pnas.2211974120 (PMC9926176; doi:10.1073/pnas.2211974120)
Supplement: Supplementary file 1 — Appendix 01 (PDF) [file pnas.2211974120.sapp.pdf]

Supplementary Information for

Landscape dynamics and diversification of the megadiverse South American freshwater fish fauna.

Fernanda A. S. Cassemiro*, James S. Albert, Alexandre Antonelli, André Menegotto, Rafael O. Wüest, Felipe Cerezer, Marco Túlio P. Coelho, Roberto E. Reis, Milton Tan, Victor Tagliacollo, Dayani Bailly, Valéria F. B. da Silva, Augusto Frota, Weferson J. da Graça, Reginaldo Ré, Telton Ramos, Anielly G. Oliveira, Murilo S. Dias, Robert K. Colwell, Thiago F. Rangel, Catherine H. Graham

Corresponding author: Fernanda A. S. Cassemiro
Email: fernandacassemiro@gmail.com

This PDF file includes:

Materials and Methods
Figures S1 to S4
Tables S1 to S7
Appendix 1
Supplementary References

Supplementary Information Text

Principal landforms controlling basin connectivity at each time interval. We relied on 1-22 to define the approximate chronology and location of the principal landscape evolution events that shaped the current drainage basins of South America and influenced the diversification of freshwater fishes. We defined, for each time interval, the possible connections among the six regions defined by the regionalization analysis (see below), as shown in Table S1.

Biogeographical Regionalization. To better understand the evolutionary history of freshwater fishes, South America was classified into six biogeographical regions (*bioregions*, hereafter). The biogeographical regionalization process starts from 490 present-day drainage basins, at level 5, according to the HydroBASINS database (https://www.hydrosheds.org/page/hydrobasins). To classify these drainage basins into biogeographical regions we used the approach proposed by 23. Considering the uncertainty in the current distribution of freshwater fishes across the 490 basins, we used 100 potential presence/absence matrices (see main text) to calculate 100 pairwise “taxonomic” dissimilarity matrices (using G. G. Simpson’s presence/absence similarity/dissimilarity index; 24, 25). We chose not to use new methods (i.e., 42) because the

index explicitly assumes that species absences a known quantity but absences, and especially double absences, are rarely reliable in biodiversity surveys. As explained in the main text, several sub-basins were poorly sampled, which led us to calculate the completeness index and employ a simulation procedure to estimate species presence/absence given the lack of adequate sample effort.

We also used phylogenetic distance among species to calculate dissimilarity matrices among basins. Then, we reduced the dimensionality of these dissimilarity matrices using NMDS. Visual inspection of the map of the first three NMDS axes (averages among 100 taxonomic NMDS), as shown in the rasterized RGB plot of Fig. S1, highlights conspicuous bioregions, which are similar in large-scale pattern, whether using taxonomic or phylogenetic similarities.

Finally, from the taxonomic dissimilarity matrices, we used K-means to cluster the 490 basins into six biogeographical regions (*bioregions*) for freshwater fishes (view main text, Fig 3). K-means clustering was repeated 100 times, applied to the 100 alternative species presence/absence matrices but generated similar clusters of basins.

Range Evolution. When running the six models using BioGeoBEARS (see *Material and Methods*), we set the parameter "max_range_size", which limits the maximum number of areas any species can occupy, to six to match the observed pattern of the maximum number of areas occupied by the freshwater fishes in our phylogeny. Additionally, we included the *null range* parameter, which allows ranges to consist of zero (0) areas, a transitional state needed for the correct computation of the log likelihood (lnL) of DEC models in BioGeoBEARS. Comparison of biogeographic models in BioGeoBEARS using AICc indicated a best fit for the BAYAREALIKE model (Table S2), which was used to all further analyses. In BAYAREALIKE, following the assumptions of the Bayesian BayArea program (26), both subset speciation (*s*) and vicariance (*v*) are given 0 weight, and widespread sympatry is allowed (unlike DEC).

Evolutionary Distinctiveness. We estimated the uniqueness of a species using the evolutionary distinctiveness measure (ED; 27) (Fig. S3). To calculate ED, the total phylogenetic diversity (PD) of a clade was split equally among its members, which estimates how much branch length, on average, is unique to each species. The ED of a species is the sum of these values for all branches from which the species is descended, to the root of the phylogeny. We calculated the average of ED by summing the branch length of all species and dividing by the total of species in each basin.

BAMM analysis. We are aware of potential limitations regarding BAMM, which has been criticized for estimating unrealistic extinction rates (28, 29). However, BAMM's proponents responded that this criticism was based on spurious premises (30). Briefly, the criticism relied on the priors used by the software, but we adjusted the priors using the latest version of BAMM package (see http://bamm-project.org), in which the prior was computed analytically for the compound Poisson process model, thus there was no need to simulate the prior distribution of the number of shifts, becoming the analysis more robust. Other problems cited by that study can be applied to most macroevolutionary methods (e.g., estimation of extinct clades) and in this sense BAMM was not considered better or worse than similar software (30). Lastly, one of the advantages of BAMM and similar methods (31-35) is that lineages can differ in their rates of speciation and extinction, that is, the rates can vary among lineages and though time. Thus, BAMM allows us to describe multiple processes that explain rates of diversification on different parts of the tree.

Analysis of Phylogenetic Endemism. We conducted a phylogenetic endemism analysis (PE; 36) as a relative measure of endemism that apportions each unit of phylogenetic diversity (PD) across the areas where it occurs. This measure identifies concentrations of taxa with spatially restricted PD (37, 38). PEA uses the branch length and the geographic range of the extant descendants of each branch on a phylogeny to sum the proportion of the geographic range of each unit of PD found in an area (each of the 490 basins). This analysis considers the geographic range of each species (39) and identifies concentrations of spatially restricted species. A time-

calibrated phylogenetic tree for a dataset of 2,523 freshwater fish species was used to calculate phylogenetic endemism as:

$$PE = \sum_{c \in C} \frac{L_c}{R_c},$$

where *C* is a phylogenetic clade spanned by minimal branches to link all taxa within one basin, and *c* is any branch between two nodes within *C*; L_c is the length of branch *c*; and R_c is the range occupied by branch *c*, defined in numbers of grid cells (36).

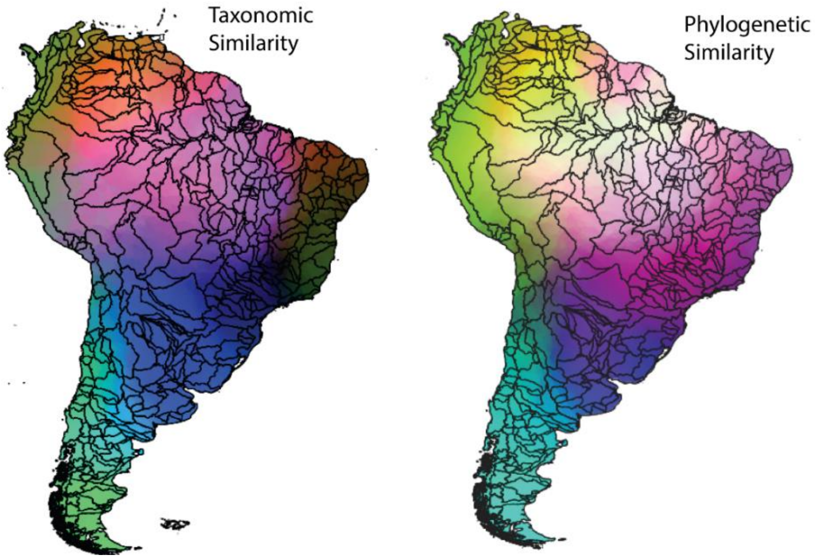

Fig. S1. RGB **i**nterpolation of the three axes of NMDS (Red axis: minimum = -0.276, maximum = 0.262; Green axis: minimum = -0.278, maximum = 0.263; Blue axis: minimum = -0.286, maximum = 0.269) based on 100 species presence/absence matrices (taxonomic similarity) and a phylogenetic matrix (phylogenetic similarity).

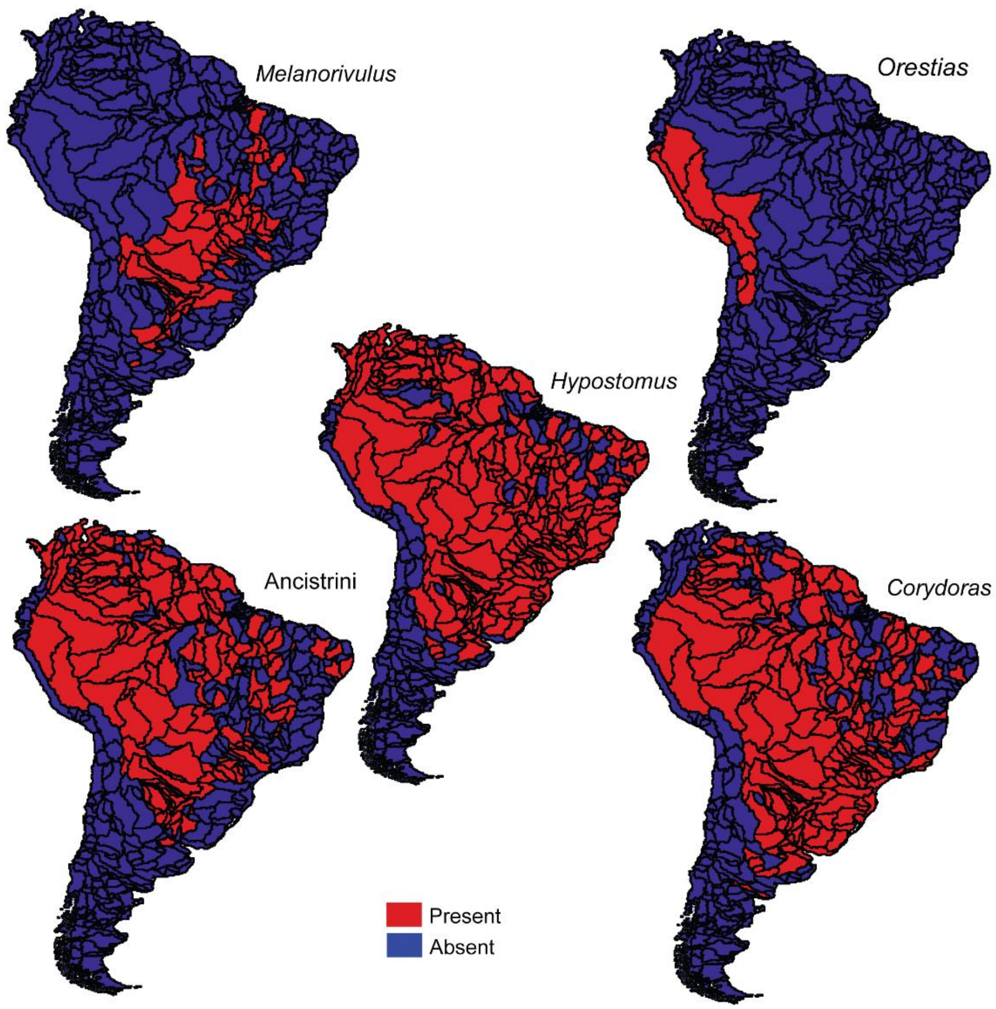

Fig. S2. Current distribution (presence and absence) of the clades that showed rapid shifts in diversification rates, as shown in Figure 2 in the main text.

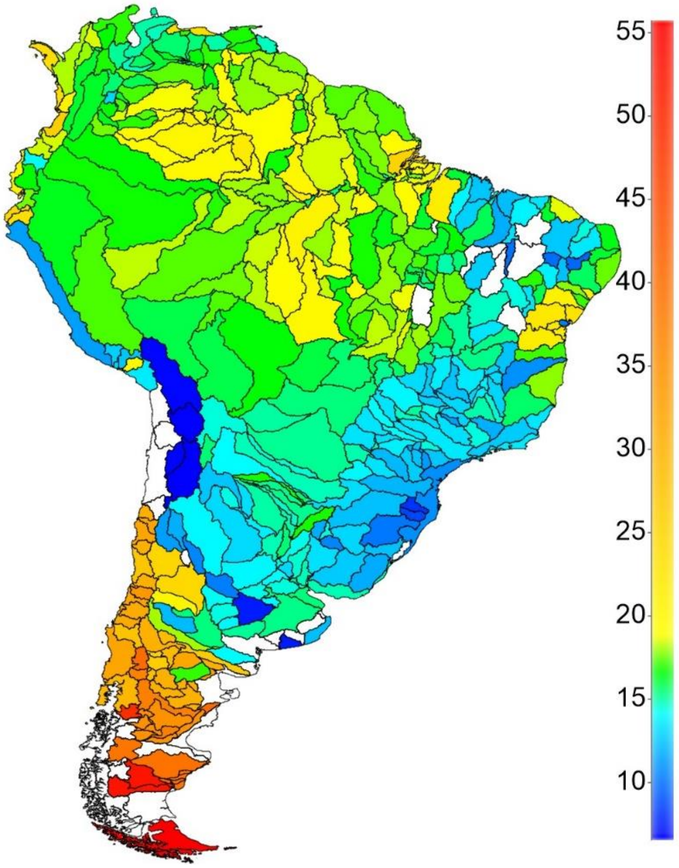

Fig. S3. Average of Evolutionary Distinctiveness (ED) per basin. ED is the proportion of the branch lengths shared with other species along the evolutionary tree and shows the amount of unique evolutionary history a lineage represents. Thus, on the map, if a species is found in a unique basin without sharing its distribution with other species, that basin will present a high ED average value. Otherwise, if a basin presents only new species (recent speciation), or has many species, the ED average value will be lower. Data for 2,523 species with phylogenetic data in 490 basins. Basins in white are data deficient.

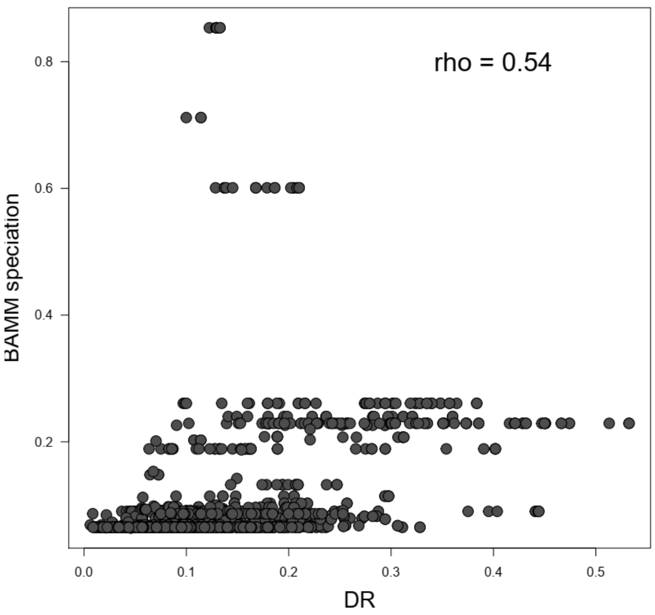

Fig. S4. Correlation between the speciation rates produced by the DR statistic and BAMM analysis.

Table S1. Paleogeographic model used for the time-stratified biogeographic analyses implemented in our range evolution models (BAYAREALIKE; DEC DIVALIKE – implemented in BioGeoBEARS package; see below). The model is built on connectivity matrices among the six bioregions, with connectivity changing through time. For any combination of two bioregions, each matrix designates whether the areas are connected (1) or disconnected (0). When two regions are connected, dispersal events and range expansions are allowed in the biogeographic reconstruction. When two bioregions are connected, an ancestor can expand its range across these regions and adjacent regions connected to them. This simple model is based on current knowledge of South American hydrogeological evolution but should be viewed as a working hypothesis to be further refined and tested. (Guianas: Orinoco, Guianas, and some trans-Andean drainages. Western-Amazonia: From Western Amazonia drainages to the area of pre-existent Purus Arch. This bioregion currently encompasses all drainage basins of the Amazon River. Eastern-Amazonia: From Purus Arch to the Atlantic Ocean, where, currently, the mouth of Amazon River is located. Pacific Coast: Most drainage systems that drain to the Pacific Coast. La Plata: Drainage systems encompassing areas currently drained by the Paraná and Paraguay Rivers, including Patagonia area. Atlantic Coast: Southeastern and Northeastern Atlantic drainage systems. All bioregions were defined according to the analysis described in the section, "Biogeographical Regionalization."

Regions	Guianas	Western-Amazonia	Eastern-Amazonia	Pacific Coast	La Plata	Atlantic Coast	Principal Landscape Events	References
**55 - 250 Ma**								
	Aquatic systems in South America were intermittently connected by multiple marine transgressions and regressions; thus drainages across the continent during this time were intermittently connected by epicontinental seaways						All regions were considered connected	1, 14
**Eocene: 55 – 33 Ma**								
**Guianas**	1	1	1	1	0	0	Sub-Andean Foreland: connected La Plata (Paraguay) and Western-Amazon	6, 15
**W-Amazonia**	1	1	1	1	1	0	Proto-Amazon: connected Eastern-Amazon and La Plata	2, 3, 6, 11
**E-Amazonia**	1	1	1	1	0	0	Low elevation in the Northern Andes	1-3
**Pacific Coast**	1	1	1	1	0	0		
**La Plata**	0	1	0	0	1	0		
**Atlantic Coast**	0	0	0	0	0	1		
**Oligocene: 33 – 23 Ma**								
**Guianas**	1	1	1	1	0	0		
**W-Amazonia**	1	1	0	1	0	0	Rise of Michicola Arch: disconnected Western-Amazon and La Plata	1, 6, 19

Regions	Guianas	Western-Amazonia	Eastern-Amazonia	Pacific Coast	La Plata	Atlantic Coast	Principal Landscape Events	References
**E-Amazonia**	1	0	1	1	0	0	Breach Gurupá Arch: when the Amazon river began flowing over the Gurupá Arch, the Central Amazon (CA) became connected to the Eastern Amazon (EA).	1, 3
**Pacific Coast**	1	1	1	1	0	0		
**La Plata**	0	0	0	0	1	1	Rift Depression indicates connection between La Plata and Southeastern Coast	1, 6-11
**Atlantic Coast**	0	0	0	0	1	1		
**Early-Middle Miocene 23 – 10 Ma**							Pebas Megawetland: diversification in Proto-Orinoco-Amazon	
**Guianas**	1	1	1	1	0	0	Pebas Megawetland extended over large areas of the modern Western Amazon and Orinoco Basins	1-5, 12-22
**W-Amazonia**	1	1	0	1	1	0	Rise of Bolivian Orocline captured, seasonally, some headwaters of La Plata drainages by the Upper Madeira River (Western-Amazon)	5, 19, 22
**E-Amazonia**	1	0	1	1	0	0		
**Pacific Coast**	1	1	1	1	0	0		
**La Plata**	0	1	0	0	1	1	Rise of Serra do Mar: partial disconnection between La Plata drainage basins from southeastern basins	6-11
**Atlantic Coast**	0	0	0	0	1	1		
**Late Miocene to Recent: 10 – 0 Ma**							Diversification in modern trans-continental Amazon	
**Guianas**	1	0	1	0	0	0	Rise of Vaupes Arch: disconnected Western Amazon and Guianas (with intermittent connection by the Casiquiare Channel)	3, 4, 17, 18
**W-Amazonia**	0	1	1	0	1	0	Rise of Northern Andes / Breach Purus Arch: connected Western and Eastern-Amazon	2, 20, 21
**E-Amazonia**	1	1	1	0	0	0	Rise of Northern Andes: disconnected Pacific Coast from Guianas and Pacific Coast from Western-Amazon.	17, 18
**Pacific Coast**	0	0	0	1	0	0		
**La Plata**	0	1	0	0	1	1		
**Atlantic Coast**	0	0	0	0	1	1		

Table S2. Models and AICc scores for three biogeographic models.

**Model**	**LnL**	***n***	***d***	***e***	***j***	**AICc**	**AICc_wt**
**DIVALIKE**	-10765	2	0.12	0.098	0	21534	0
**DEC**	-10150	2	0.086	0.073	0	20305	0
**BAYAREALIKE**	-8059	2	0.037	0.075	0	16123	1

Table S3. Average number of dispersal events (from 20 simulations) estimated by Biogeographical Stochastic Mapping analysis, using the maximum likelihood version of Dispersal-Vicariance analysis (**DIVALIKE**; BioGeoBEARS package). Bold numbers represent the highest values of dispersal from (lines) one region to (columns) another region.

Regions From/To	Guianas	W-Amazonia	E-Amazonia	Pacific Coast	La Plata	Atlantic Coast	Total	%
**Guianas**	-	420.7	**650**	426.3	50	51	1598	16.34
**W-Amazon**	453.3	-	**668.7**	545.1	932.6	52.43	2652.13	**27.12**
**E-Amazon**	**647**	577.4	-	491	49.86	50.71	1815.97	**18.57**
**Pacific Coast**	409.6	495.4	**551.4**	-	52.29	59.43	1568.12	16.04
**La Plata**	55.29	512	44.86	62.86	-	**825.9**	1500.91	15.35
**Atlantic Coast**	70.57	53.29	47.86	64.14	**407.7**	-	643.56	6.58
						**Total**	9778.7	100

Table S4. Average number of dispersal events (from 20 simulations) estimated by Biogeographical Stochastic Mapping analysis, using the Dispersal-Extinction-Cladogenesis model (**DEC**; BioGeoBEARS package). Bold numbers represent the highest values of dispersal from (lines) one region to (columns) another region.

Regions From/To	Guianas	W-Amazonia	E-Amazonia	Pacific Coast	La Plata	Atlantic Coast	Total	%
**Guianas**	-	298	**415**	254	30.5	50.5	1048	14.83
**W-Amazon**	311.5	-	501.5	436.5	**655.5**	46	1951	**27.62**
**E-Amazon**	**453**	379	-	385	37.5	48.5	1303	**18.44**
**Pacific Coast**	322.5	398	**472**	-	37	46	1275.5	18.05
**La Plata**	43	326.5	38.5	47.5	-	**615**	1070.5	15.15
**Atlantic Coast**	60.5	29	26.5	29	**272**	-	417	5.90
						**Total**	7065	100

Table S5. Average number of dispersal events (from 20 simulations) estimated by Biogeographical Stochastic Mapping analysis, using the Bayesian Biogeographical Inference model (**BAYAREALIKE**; BioGeoBEARS package). Bold numbers represent the highest values of dispersal from (lines) one region to (columns) another region.

Regions From/To	Guianas	W-Amazonia	E-Amazonia	Pacific Coast	La Plata	Atlantic Coast	Total	%
**Guianas**	-	145.6	**205.8**	132	24.4	27.3	535.1	14.3
**W-Amazon**	182.7	-	228.4	198.1	**340.7**	26.95	976.85	**26.2**
**E-Amazon**	**248.4**	237.6	-	158.5	22.5	28.85	695.85	**18.7**
**Pacific Coast**	178.5	197.2	**217**	-	23.55	29.25	645.5	17.3
**La Plata**	24.25	183.6	19.35	20.55	-	**320.2**	567.95	15.2
**Atlantic Coast**	27.65	22.5	23.05	23.15	**213.3**	-	309.65	8.3
						**Total**	3730.9	100

Table S6. Proportion of dispersal events (%) among the six regions considering the total number of dispersal events, (**BAYAREALIKE**). Bold numbers represent the highest proportions of dispersal events from the total of dispersal events (3,730.9) among all regions. See also Table S3.

Regions From/To	Guianas	W-Amazonia	E-Amazonia	Pacific Coast	La Plata	Atlantic Coast	% of total dispersal events
**Guianas**	-	3.9	5.5	3.5	0.7	0.7	14.3
**W-Amazon**	4.9	-	6.1	5.3	9.1	0.7	**26.2**
**E-Amazon**	6.7	6.4	-	4.2	0.6	0.8	**18.7**
**Pacific Coast**	4.8	5.3	5.8	-	0.6	0.8	17.3
**La Plata**	0.6	4.9	0.5	0.6	-	8.6	15.2
**Atlantic Coast**	0.7	0.6	0.6	0.6	5.7	-	8.3

Table S7. Proportion of dispersal events (%) among six regions considering the total number of dispersal events from each region to another (**BAYAREALIKE**). Bold numbers represent the highest proportions of dispersal events from (lines) one region to (columns) another region.

Regions From/To	Guianas	W-Amazonia	E-Amazonia	Pacific Coast	La Plata	Atlantic Coast	Total (%)
**Guianas**	0.0	**27.2**	**38.5**	24.7	4.6	5.1	100
**W-Amazon**	18.7	0.0	**23.4**	20.3	**34.9**	2.8	100
**E-Amazon**	**35.7**	**34.1**	0.0	22.8	3.2	4.1	100
**Pacific Coast**	27.7	**30.5**	**33**.6	0.0	3.6	4.5	100
**La Plata**	4.3	32.3	3.4	3.6	0.0	**56.4**	100
**Atlantic Coast**	8.9	7.3	7.4	7.5	**68.9**	0.0	100

SI References

1. J. G. Lundberg et al. The stage for Neotropical Fish Diversification: A History of Tropical South American Rivers. *In Phylogeny and Classification of Neotropical Fishes*, M. Malabarba, R. E. Reis, R. P. Vari, Z. M. Lucena, C. A. S. Lucena, Eds. (Edipucrs, 1998), pp. 13–48.
2. C. M. Hoorn *et al*., Amazonia through time: Andean uplift climate change, landscape evolution and biodiversity. *Science* **330**, 927–31 (2010).
3. C. Hoorn, F. P. Wesselingh, Eds., *Amazonia—landscape and species evolution: a look into the past*. (Wiley-Blackwell, 2010).
4. J. S. Albert, R. E. Reis, Eds., *Historical biogeography of Neotropical freshwater fishes* (University of California Press, 2011).
5. V. A. Tagliacollo, F. F. Roxo, S. M. Duke-Sylvester, C. Oliveira, J. S. Albert, Biogeographical signature of river capture events in Amazonian lowlands. *J. Biogeogr*. **42**, 2349–2362 (2015).
6. N. Cogné, K. Gallagher, P. R. Cobbold, C. Riccomini, C. Gautheron, Post-breakup tectonics in southeast Brazil from thermochronological data and combined inverse-forward thermal history modeling. *J. Geophys. Res. Solid Earth* **117**, 1–16 (2012).
7. A. C. Ribeiro, Tectonic history and the biogeography of the freshwater fishes from the coastal drainages of eastern Brazil: an example of faunal evolution associated with a divergent continental margin. *Neotrop. Ichthyol.* **4**, 225–246 (2016).
8. C. A. Tello Saenz, *et al.*, Recognition of Cretaceous, Paleocene, and Neogene tectonic reactivation through apatite fission-track analysis in Precambrian areas of southeast Brazil: association with the opening of the south Atlantic Ocean. *Journal of South American Earth Sciences* **15**, 765–774 (2003).
9. D. H. de Souza, F. M. Stuart, Á. Rodés, F. N. Pupim, P. C. Hackspacher, Controls on the erosion of the continental margin of southeast Brazil from cosmogenic 10Be in river sediments. *Geomorphology* **330**, 163–176 (2019).
10. D. H. Souza, P. C. Hackspacher, B. V. Silva, M. C. Siqueira-Ribeiro, S. T. Hiruma, Temporal and spatial denudation trends in the continental margin of southeastern Brazil. *Journal of South American Earth Sciences* **105**, 102931 (2021).
11. A. C. Ribeiro, Tectonic history and the biogeography of the freshwater fishes from the coastal drainages of eastern Brazil: an example of faunal evolution associated with a divergent continental margin. Neotrop. Ichthyol. 4, 225–246 (2016).
12. J. S. Albert, P. Val, C. Hoorn, The changing course of the Amazon River in the Neogene: center stage for Neotropical diversification. *Neotrop. Ichthyol*. **16**, e180033 (2018).
13. T. C. Bicudo, V. Sacek, R. P. Almeida, J. M. Bates, C. C. Ribas, Andean tectonics and mantle dynamics as a pervasive influence on Amazonian ecosystem. *Sci. Rep.* **9**, 16879 (2019).
14. R. Bernal, *et al.*, Could coastal plants in western Amazonia be relicts of past marine incursions? *J. Biogeogr.* **46**, 1749–1759 (2019).
15. J.G. Lundberg, M. H. Sabaj-Pérez, W. M. Dahdul, O. A. Aguilera. "The Amazonian Neogene fish fauna", in *Amazonia—landscape and species evolution: a look into the past*, C. Hoorn, F. P. Wesselingh, Eds. (Wiley-Blackwell, 2010), pp. 281–301.
16. J. S. Albert, P. Val, C. Hoorn, The changing course of the Amazon River in the Neogene: center stage for Neotropical diversification. *Neotrop. Ichthyol*. **16**, e180033 (2018).
17. W. G. R. Crampton, Miocene tectonism and the separation of cis- and trans-Andean river basins: Evidence from Neotropical fishes *J. South Amer. Earth Sci,* 21, 14–27 (2006).
18. Mora, A., Villagómez, D., Parra, M., Caballero, V. M., Spikings, R., Horton, B. K., ... & Arias-Martínez, J. P., Late Cretaceous to Cenozoic uplift of the northern Andes: Paleogeographic implications. *The Geology of Colombia*, **3**, 89–121 (2020).
19. M. Brea, A. F. Zucol, "The Paraná-Paraguay Basin: Geology and Paleoenvironments" in J. S. Albert, R. E. Reis, Eds. (University of California Press, 2011), pp. 69–87.

20. C. Hoorn, *et al*., The Amazon at sea: Onset and stages of the Amazon River from a marine record, with special reference to Neogene plant turnover in the drainage basin. *Glob. Planet. Change* **153**, 51–65 (2017).
21. J. S. Albert, D. R. Schoolmaster JR., V. Tagliacollo, S. M. Duke-Sylvester, Barrier Displacement on a Neutral Landscape: Toward a Theory of Continental Biogeography. *Syst. Biol.* **66**, 167–182 (2017).
22. T. P. Carvalho, J. S. Albert, "The Amazon-Paraguay divide" in *Historical biogeography of Neotropical freshwater fishes*, J. S. Albert, R. E. Reis, Eds. (University of California Press, 2011), pp. 193–202.
23. H. Kreft, H., W. Jetz, A framework for delineating biogeographical regions based on species distributions. *J. Biogeogr.* **37**, 2029–2053 (2010).
24. Simpson, G. G. Mammals and the nature of continents. *Am. J. Sci.* **241**:1–31 (1943).
25. Koleff, P., K. E. Gaston, and J. J. Lennon. Measuring beta diversity for presence–absence data. *J. Animal Ecol*. **72**:367–382 (2003).
26. M. J. Landis, N. J. Matzke, B. R. Moore, J. P. Huelsenbeck, Bayesian Analysis of Biogeography when the Number of Areas is Large. *Systematic Biology* **62**, 789–804 (2013).
27. N. J. B., Isaac, S. T. Turvey, B. Collen, C. Waterman. J. E. M., Baillie, Mammals on the EDGE: conservation priorities based on threat and phylogeny. *PLoS ONE*, **2**, e296D (2007).
28. B. R. Moore, S. Höhna, M. R. May, B. Rannala, J. P. Huelsenbeck, Critically evaluating the theory and performance of Bayesian analysis of macroevolutionary mixtures. *Proc. Natl. Acad. Sci.* **113**, 9569–9574 (2016).
29. A. L. S. Meyer, J. J. Wiens, Estimating diversification rates for higher taxa: BAMM can give problematic estimates of rates and rate shifts. *Evolution (N. Y).* **72**, 39–53 (2018).
30. P. O. Title, D. L. Rabosky, Tip rates, phylogenies and diversification: What are we estimating, and how good are the estimates? *Methods Ecol. Evol.* **10**, 821–834 (2019).
31. H. Morlon, *et al.*, <scp>RPANDA</scp>: an R package for macroevolutionary analyses on phylogenetic trees. *Methods Ecol. Evol.* **7**, 589–597 (2016).
32. S. Höhna, *et al.*, RevBayes: Bayesian Phylogenetic Inference Using Graphical Models and an Interactive Model-Specification Language. *Syst. Biol.* **65**, 726–736 (2016).
33. O. Maliet, F. Hartig, H. Morlon, A model with many small shifts for estimating species-specific diversification rates. *Nat. Ecol. Evol.* **3**, 1086–1092 (2019).
34. J. Barido-Sottani, *et al.*, Ignoring Fossil Age Uncertainty Leads to Inaccurate Topology and Divergence Time Estimates in Time Calibrated Tree Inference. *Front. Ecol. Evol.* **8** (2020).
35. C. B. Beeravolu, F. L. Condamine, An Extended Maximum Likelihood Inference of Geographic Range Evolution by Dispersal, Local Extinction and Cladogenesis. *bioRxiv* **038695** (2016).
36. D. Rosauer, S. W. Laffan, M. D. Crisp, S. C. Donnellan, L. G. Cook, Phylogenetic endemism: a new approach for identifying geographical concentrations of evolutionary history. *Mol. Ecol.* **18**, 4061–4072 (2009).
37. F. D. Faith, C. A. M. Reid, J. Hunter, Integrating Phylogenetic Diversity, Complementarity, and Endemism. *Conserv. Biol.* **18**, 255–261 (2004).
38. M. W. Cadotte, T. Jonathan Davies, Rarest of the rare: advances in combining evolutionary distinctiveness and scarcity to inform conservation at biogeographical scales. *Divers. Distrib.* **16**, 376–385 (2010).
39. M. Crisp, S. Laffan, H. Linder, A. Monro Endemism in the Australian flora. *J. Biogeogr.*, **28**, 183–198 (2001).
40. L. C. Hughes, *et al.*, Comprehensive phylogeny of ray-finned fishes (Actinopterygii) based on transcriptomic and genomic data. *Proc. Natl. Acad. Sci.* **115**, 6249–6254 (2018).
41. J. P. Fontenelle, F. Portella Luna Marques, M. A. Kolmann, N. R. Lovejoy, Biogeography of the neotropical freshwater stingrays (Myliobatiformes: Potamotrygoninae) reveals effects of continent-scale paleogeographic change and drainage evolution. *Journal of Biogeography* **48**, 1406–1419 (2021).
42. K. P. Mainali, E. Slud, M. C. Singer, W.F. Fagan, A better index for analysis of co-occurrence and similarity. *Sci. Adv.*, **8**, p.eabj9204 (2022)
